# Molecular taxonomy and spatial organization define neuronal subtypes in the mouse inferior colliculus

**DOI:** 10.1016/j.isci.2026.116484

**Published:** 2026-06-23

**Authors:** Mengting Liu, Qi Hu, Wenhao Feng, Qi Guo, Tianyu Ma, Xiang Li, Qi Meng, Shijia Xu, Jueqi Li, Tianhong Zhang, Huaizhang Shi

**Affiliations:** 1 First Affiliated Hospital of Harbin Medical University, Harbin, Heilongjiang 150001, China; 2 Department of Otorhinolaryngology Head and Neck Surgery, First Affiliated Hospital of Harbin Medical University, Harbin, China; 3 Shanghai Key Laboratory of Gene Editing and Cell Therapy for Rare Diseases, Fudan University, Shanghai 200031, China; 4 Department of Science and Technology, Second Affiliated Hospital of Harbin Medical University, Harbin, China; 5 Department of Neurosurgery, First Affiliated Hospital of Harbin Medical University, Harbin, China

**Keywords:** molecular neuroscience, cell biology, transcriptomics

## Abstract

The inferior colliculus (IC) is a critical auditory midbrain hub that processes sound localization and multisensory behaviors. Using single-nucleus RNA sequencing, we identify 14 transcriptionally distinct neuronal subtypes in the adult mouse IC. *In situ* sequencing and spatial mapping confirm these organizational patterns. Excitatory subtypes show region-specific spatial enrichment across the central, dorsal, and external cortices, whereas inhibitory subtypes are more diffusely distributed. A somatostatin-expressing (*Sst*^*+*^) neuronal population is predominantly excitatory but exhibits regional heterogeneity, with a subset co-expressing *Gad2*. Sub-clustering resolves *Sst*^*+*^ neurons into five molecularly distinct subtypes. Complementary to the Allen Brain Cell Atlas, this work provides an IC-focused molecular and spatial resource for studying neuronal diversity and cell-type-specific circuit organization in the auditory midbrain.

## Highlights

• Single-nucleus RNA-seq identifies 14 neuronal subtypes in mouse IC
• Excitatory IC subtypes show region-specific spatial enrichment
• *Sst*^+^ IC neurons are mostly excitatory yet transcriptionally heterogeneous
• *In situ* sequencing links molecular identity to IC anatomical organization

## Graphical abstract

**Figure:**
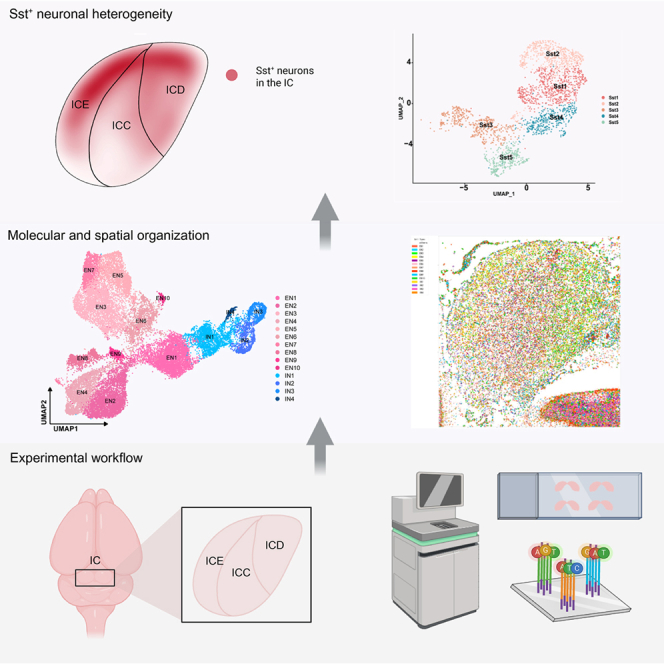

## Introduction

The auditory midbrain, or inferior colliculus (IC), serves as a critical hub in the central auditory pathway. It integrates ascending inputs from numerous lower auditory nuclei, including the cochlear nucleus, superior olivary complex, and nuclei of the lateral lemniscus, along with descending projections from the auditory thalamus and cortex.^1^^,^^2^ As an essential center for acoustic signal processing,^3^^,^^4^ the IC mediates auditory-guided behaviors such as sound localization,^5^ frequency discrimination,^6^ innate sound-evoked responses,^7^^,^^8^^,^^9^ and the detection of environmental cues to guide adaptive motor output.^10^ Recent studies have also demonstrated that IC neurons integrate non-auditory signals related to movement, exhibiting activity modulated by locomotion even in the absence of auditory input, indicating a broader role in auditory-motor integration.^11^ Beyond its role in normal hearing, the IC has been implicated in disorders such as tinnitus^12^^,^^13^ and autism spectrum disorder,^14^^,^^15^ underscoring its importance in both physiological and pathological contexts.^1^

Refining the classification of IC neurons is essential to understanding their cellular properties and functional roles. Historically, neuronal categorization in the IC has relied on diverse methodologies: early morphological studies distinguished disc-shaped and stellate cells using Golgi staining,^16^ electrophysiological recordings classified neurons based on their firing patterns,^17^ and neurochemical approaches later separated them into glutamatergic excitatory and GABAergic inhibitory types based on neurotransmitter content.^18^^,^^19^ However, these conventional strategies often capture isolated aspects of neuronal identity and lack a unified framework that integrates morphological, physiological, and molecular characteristics.

Recent advances in genetic engineering and viral tracing have enabled the classification of IC neurons using molecular markers such as cholecystokinin (*Cck*),^20^ neuropeptide Y (*Npy*),^21^^,^^22^^,^^23^^,^^24^ vasoactive intestinal peptide (*Vip*),^24^^,^^25^^,^^26^^,^^27^^,^^28^ parvalbumin (*Pv*),^29^ and somatostatin (*Sst*).^30^ These markers correlate closely with the functional and anatomical properties of IC neurons. For instance, *Cck*-positive neurons are predominantly excitatory,^20^ while *Npy*-positive cells are mainly inhibitory and often display sustained firing patterns.^21^ Moreover, *Pv*- and *Sst*-expressing neurons are localized to distinct subregions of the IC and project to different subdivisions of the auditory thalamus.^30^ Despite these advances, current molecular classifications largely rely on single-gene labels, grouping neurons into broad categories rather than providing a refined, multi-parametric definition of cell types.^31^

Single-nucleus RNA sequencing (snRNA-seq) and spatial transcriptomics offer powerful approaches to address this limitation by enabling unbiased, genome-wide transcriptional profiling at cellular resolution.^32^^,^^33^^,^^34^^,^^35^ Compared with single-cell RNA sequencing (scRNA-seq), snRNA-seq is particularly well suited for profiling adult neural tissue as it reduces dissociation-induced stress artifacts and captures fragile neuronal populations more comprehensively.^36^^,^^37^ However, snRNA-seq may detect fewer genes, particularly cytoplasmic transcripts, than whole-cell scRNA-seq. Therefore, snRNA-seq and scRNA-seq should be viewed as complementary approaches. In this study, we applied snRNA-seq to characterize neuronal diversity in the adult mouse IC and identified 14 transcriptionally distinct neuronal subtypes. To examine their anatomical distribution, we selected subtype-enriched marker genes and mapped them across IC subregions using multiplexed ISS. By integrating transcriptomic classification with spatial mapping, this study provides an IC-focused molecular and spatial resource for examining excitatory and inhibitory neuronal organization in the auditory midbrain.

Recent studies have suggested that *Sst*^+^ neurons in the IC form distinct tectothalamic pathways and exhibit subregion-specific projection patterns.^30^^,^^38^ However, their molecular heterogeneity and functional roles remain poorly understood. In this study, we demonstrate that *Sst*^+^ neurons in the IC predominantly exhibit an excitatory molecular phenotype, which contrasts with their traditionally known inhibitory role in other brain regions. Through sub-clustering, we resolve *Sst*^+^ neurons into five transcriptionally distinct subtypes (Sst1-Sst5). Among these, Sst1, Sst2, Sst4, and Sst5 are almost exclusively excitatory, while Sst3 contains a notable inhibitory component (35.2% inhibitory-derived). Pathway enrichment analyses generated hypotheses regarding subtype-associated molecular programs related to neuromodulatory signaling, synaptic plasticity, and reward-associated pathways. These findings provide a molecular framework for future studies investigating how distinct *Sst*^+^ subpopulations may participate in auditory and multisensory circuits, while also revealing a more complex, heterogeneous role for *Sst*^+^ neurons in the IC than previously thought.

This study establishes an integrated molecular and spatial atlas of the IC, providing a foundational resource for understanding cell-type-specific circuits involved in auditory processing and behavior. Additionally, it offers insights that could inform therapeutic strategies for IC-related disorders.

## Results

### Single-nucleus RNA sequencing confirms seven major cell types in the mouse inferior colliculus

**Figure 1:**
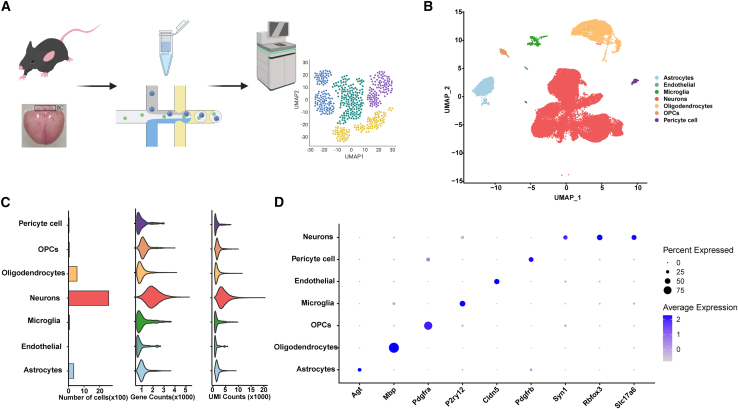
snRNA-seq identifies major cell types in the mouse inferior colliculus (A) Experimental workflow illustrating IC dissection from 9 mice, pooled into three independent samples (three mice per sample), followed by nuclei isolation, snRNA-seq using the 10x Genomics platform, and downstream bioinformatic analysis. (B) UMAP projection of 36,765 high-quality nuclei, colored by seven major transcriptionally defined cell types. (C) Quantification of the seven major cell types in the IC. Left: total cell counts per cell type. Middle: number of detected genes per cell. Right: number of UMIs per cell. Bar plot shows relative abundance of each cell type. (D) Dot plot showing expression of canonical marker genes used for cell-type annotation. Dot size indicates the percentage of cells expressing each gene, and color represents average expression level.

To construct a high-resolution cellular atlas of the auditory midbrain, we performed large-scale snRNA-seq on the mouse IC. IC tissues were collected from nine 2-month-old C57BL/6J mice and processed as three independent pooled samples (each comprising tissue from three mice) to ensure sufficient nuclei yield and library quality (Figure 1A). Nuclei were isolated and sequenced using the 10x Genomics Chromium platform.

After stringent quality control filtering (see STAR Methods), we retained 36,765 high-confidence nuclei for downstream analysis. Unsupervised clustering using principal component analysis and graph-based clustering identified seven major transcriptionally distinct cell populations, visualized by uniform manifold approximation and projection (UMAP) (Figure 1B).

Quantification of cellular composition showed that neurons constituted the predominant population, followed by oligodendrocytes and astrocytes. Smaller proportions included oligodendrocyte precursor cells (OPCs), microglia, endothelial cells, and pericytes (Figure 1C). These proportions are consistent with previous reports of cellular composition in the mammalian midbrain.

Cell-type identities were assigned based on the expression of well-established canonical marker genes (Figure 1D). Neurons were defined by robust expression of *Syn1* and *Rbfox3* (NeuN). The oligodendrocyte lineage was clearly resolved, with mature oligodendrocytes expressing *Mbp* and OPCs marked by *Pdgfra*. Astrocytes were identified by *Agt*, microglia by *P2ry12*, endothelial cells by *Cldn5*, and pericytes by *Pdgfrb*.

These results define seven major IC cell types with clear transcriptional separation, providing a reliable foundation for subsequent neuronal subtype analysis.

### Molecular subclassification of excitatory and inhibitory neuronal populations in the IC

**Figure 2:**
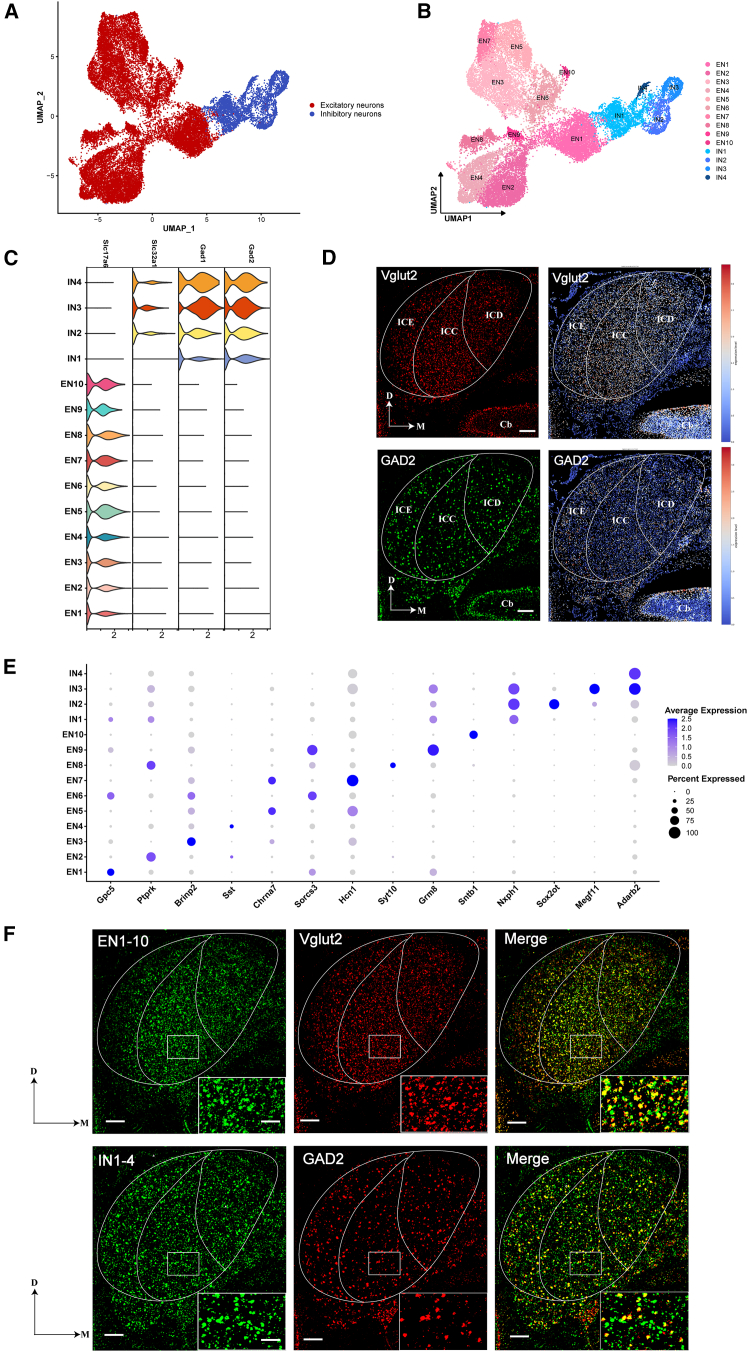
Subclassification and spatial mapping of excitatory and inhibitory neuronal subtypes in the IC (A) UMAP visualization showing segregation of IC neurons into excitatory and inhibitory populations. (B) Subtype-level UMAP of 10 excitatory (EN1-EN10) and 4 inhibitory (IN1-IN4) clusters obtained through transcriptome-based sub-clustering. (C) Expression profiles of pan-neuronal markers: excitatory neurons uniformly express *Slc17a6* (*Vglut2*), while inhibitory neurons express *Slc32a1* (*Vgat*), *Gad*1, and *Gad2*. (D) Left: ISS images showing the spatial expression patterns of *Vglut2* and *Gad2* in IC sections. Right: Heatmaps representing the relative expression density of *Vglut2* and *Gad2* across IC subregions (ICC, ICD, and ICE), with warmer colors (red) indicating higher expression and cooler colors (blue) indicating lower expression. (E) Dot plot of subtype-specific marker genes (e.g., *Ptprk* for EN2, *Sntb1* for EN10, *Grm8*/*Sorcs3* for EN9). Dot size indicates expression percentage, color represents average expression level. (F) Global validation of neurotransmitter identity for transcriptomically defined subtypes. Top: composite image of all 10 excitatory subtype markers (*Gpc5*, *Ptprk*, *Brinp2*, *Sst*, *Chrna7*, *Sorcs3*, *Hcn1*, *Syt10*, *Grm8*, and *Sntb1*; green) overlaid with the pan-excitatory marker *Vglut2* (red). Bottom: composite image of all 4 inhibitory subtype markers (*Nxph1*, *Sox2ot*, *Megf11*, and *Adarb2*; green) overlaid with the pan-inhibitory marker *Gad2* (red). Insets in the lower right corners show 2× magnified views of the boxed regions, highlighting co-localization of subtype markers with the corresponding neurotransmitter markers at single-cell resolution. Scale bars: 50 μm (main panels) and 25 μm (insets).

Given the predominance of neurons in the IC, we performed further sub-clustering to resolve their transcriptional diversity. Neurons were first segregated by neurotransmitter phenotype into excitatory (*Vglut2*) and inhibitory *(Vgat*/*Gad1*/*Gad2*) populations, as visualized by UMAP (Figure 2A). Unsupervised clustering subsequently identified 10 transcriptionally distinct excitatory (EN1-EN10) and 4 inhibitory (IN1-IN4) subtypes (Figure 2B). Expression profiling of pan-neurotransmitter markers further supported this classification (Figure 2C). *Slc17a6* (*Vglut2*) was broadly enriched across all excitatory subtypes, whereas *Slc32a1* (*Vgat*), *Gad1*, and *Gad2* were preferentially expressed in inhibitory subtypes, confirming the robustness of the excitatory-inhibitory segregation.

To examine the transcriptional distinctness of each neuronal subtype, we generated a heatmap of the top differentially expressed genes across EN1-EN10 and IN1-IN4 (Figure S1). This analysis revealed clear and subtype-specific gene expression patterns, with limited overlap between clusters, supporting the robustness and separability of the identified neuronal subtypes.

To further define the molecular identity of each subtype, we examined representative subtype-enriched marker genes (Figure 2E). Some subtypes could be distinguished by highly specific single markers, such as *Gpc5* for EN1, *Ptprk* for EN2, *Brinp2* for EN3, *Sst* for EN4, *Hcn1* for EN7, *Syt10* for EN8, *Sntb1* for EN10, *Nxph1* for IN1, *Sox2ot* for IN2, *Megf11* for IN3, and *Adarb2* for IN4. Other subtypes were better defined by relative enrichment patterns across multiple markers, such as *Sorcs3* and *Grm8* among excitatory populations.

We next used ISS to examine the spatial distribution of the pan-excitatory marker *Vglut2* and the pan-inhibitory marker *Gad2* in IC sections (Figure 2D). Both markers were broadly distributed across IC subdivisions and showed intermingled spatial patterns without sharp regional segregation. Density maps further indicated that excitatory and inhibitory neurons are widely represented throughout the central nucleus (ICC), dorsal cortex (ICD), and external cortex (ICE) (Figure 2D).

To validate the neurotransmitter identity of the transcriptomically defined subtypes *in situ*, we generated composite ISS images by merging all 10 excitatory subtype markers or all 4 inhibitory subtype markers and comparing them with *Vglut2* or *Gad2*, respectively (Figure 2F). The merged excitatory markers showed strong overlap with *Vglut2*, whereas the merged inhibitory markers overlapped with *Gad2*, confirming the consistency between transcriptomic classification and spatially detected neurotransmitter identity. These findings support a molecular framework in which IC neurons can be divided into distinct excitatory and inhibitory subtypes, with spatial organization validated *in situ*.

### Molecular characterization of IC neuronal subtypes

**Figure 3:**
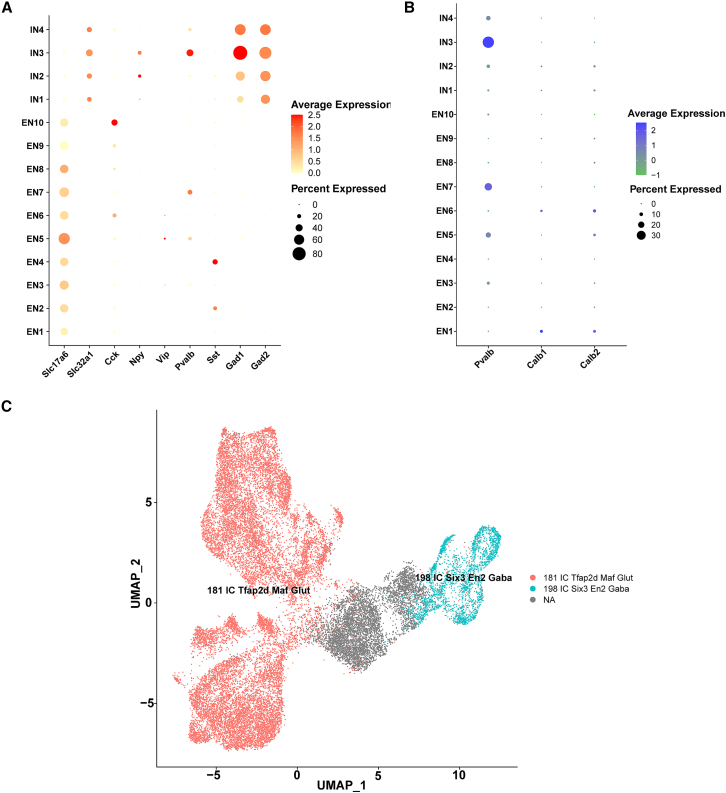
Molecular characterization of IC neuronal subtypes and comparison with the Allen brain cell atlas (A) Dot plot showing expression of established molecular markers across excitatory (EN1-EN10) and inhibitory (IN1-IN4) neuronal subtypes. Markers include *Slc17a6* (*Vglut2*), *Slc32a1* (*Vgat*), *Gad1*, *Gad2*, *Cck*, *Npy*, *Vip*, *Pvalb*, and *Sst*. Dot size indicates the percentage of cells expressing each gene, and color indicates average expression level. (B) Expression of calcium-binding proteins across neuronal subtypes. Dot plot showing distribution of *Pvalb*, *Calb1*, and *Calb2* across EN1-EN10 and IN1-IN4 populations. (C) Comparison with the Allen brain cell (ABC) Atlas using reference mapping. UMAP showing projection of Allen IC subclass annotations onto our dataset. Cells are colored according to assigned ABC subclasses, including glutamatergic and GABAergic reference types, with unassigned cells labeled as “NA”.

To assess our transcriptomic classification, we examined the expression of established molecular markers across IC neuronal subtypes (Figure 3A). As expected, *Slc17a6* (*Vglut2*) was broadly enriched across excitatory subtypes, whereas inhibitory markers (*Vgat*, *Gad1*, and *Gad2*) were preferentially expressed in inhibitory clusters, confirming the neurotransmitter-based segregation of excitatory and inhibitory neuronal populations.

Notably, *Sst* was enriched in excitatory subtypes EN2 and EN4, while *Gad1* and *Gad2* remained largely restricted to inhibitory clusters, indicating that the major *Sst*-associated populations identified at this stage are molecularly distinct from canonical GABAergic neurons. In agreement with previous studies, *Cck* expression was restricted to excitatory neurons and was most prominent in EN6 and EN10, whereas *Npy* preferentially marked inhibitory populations, particularly IN2 and IN3. *Vip* was detected primarily in EN5, albeit at relatively low levels. In contrast, *Pvalb* exhibited a more complex expression pattern, with enrichment in both inhibitory (IN3) and excitatory (EN7, and to a lesser extent EN5) subtypes, indicating that it marks functionally distinct neuronal populations rather than a single neurotransmitter-defined class. Together, these marker expression patterns provide independent molecular support for our subtype assignments and refine the identities of IC neuronal populations.

We next examined the distribution of calcium-binding proteins, which are associated with neuronal physiological properties and circuit specialization (Figure 3B). *Pvalb* again showed strong enrichment in a restricted subset of neuronal populations, primarily IN3 and EN7. In comparison, *Calb1* and *Calb2* exhibited more selective expression patterns: *Calb1* was detected mainly in EN6, whereas *Calb2* was enriched in EN6 and present at lower levels in a subset of inhibitory neurons. These results further highlight the molecular heterogeneity of IC neurons and provide an additional layer of subtype characterization beyond the broad excitatory-inhibitory classification.

### Comparison with the Allen brain cell atlas

To benchmark our IC neuronal taxonomy against a publicly available reference, we compared our snRNA-seq dataset with the Allen brain cell (ABC) Atlas using a reference-mapping approach. Specifically, we applied MapMyCells to project ABC IC subclass annotations onto our dataset. This analysis was based on reference mapping at the subclass level.

The mapping results revealed strong concordance at the level of major neuronal classes. The Allen glutamatergic subclass (”181 IC Tfap2d Maf Glut”) predominantly aligned with excitatory neuronal populations in our dataset, whereas the GABAergic subclass (”198 IC Six3 En2 Gaba”) mapped primarily to inhibitory populations (Figure 3C). These findings confirm that our classification of IC neurons into excitatory and inhibitory groups is consistent with the ABC reference framework.

Despite this agreement at the broad class level, differences were observed when comparing our IC-focused subtypes with ABC subclass-level annotations. Our dataset resolved 14 transcriptionally distinct neuronal subtypes (EN1-EN10 and IN1-IN4), each defined by specific marker gene combinations. In contrast, the ABC atlas primarily represents IC neurons at the level of broad glutamatergic and GABAergic subclasses. Consistent with this difference in granularity, a substantial fraction of cells in our dataset could not be confidently assigned to existing Allen IC subclasses and were labeled as “NA” in the reference mapping (Figure 3C). We interpret this partial mismatch cautiously. Because our comparison was based on subclass-level reference mapping rather than full integration across all ABC taxonomy levels, cells labeled as “NA” should not be interpreted as evidence of distinct biological identities. Instead, these unmapped cells may reflect differences in mapping confidence, taxonomic level, sampling strategy, and technical features of scRNA-seq versus snRNA-seq. While the ABC Atlas provides a comprehensive brain-wide reference, our IC-focused dataset captures additional subtype-level heterogeneity and provides spatial validation within the IC, thereby serving as a complementary resource.

### Spatially resolved organization of molecularly defined IC neuronal subtypes

**Figure 4:**
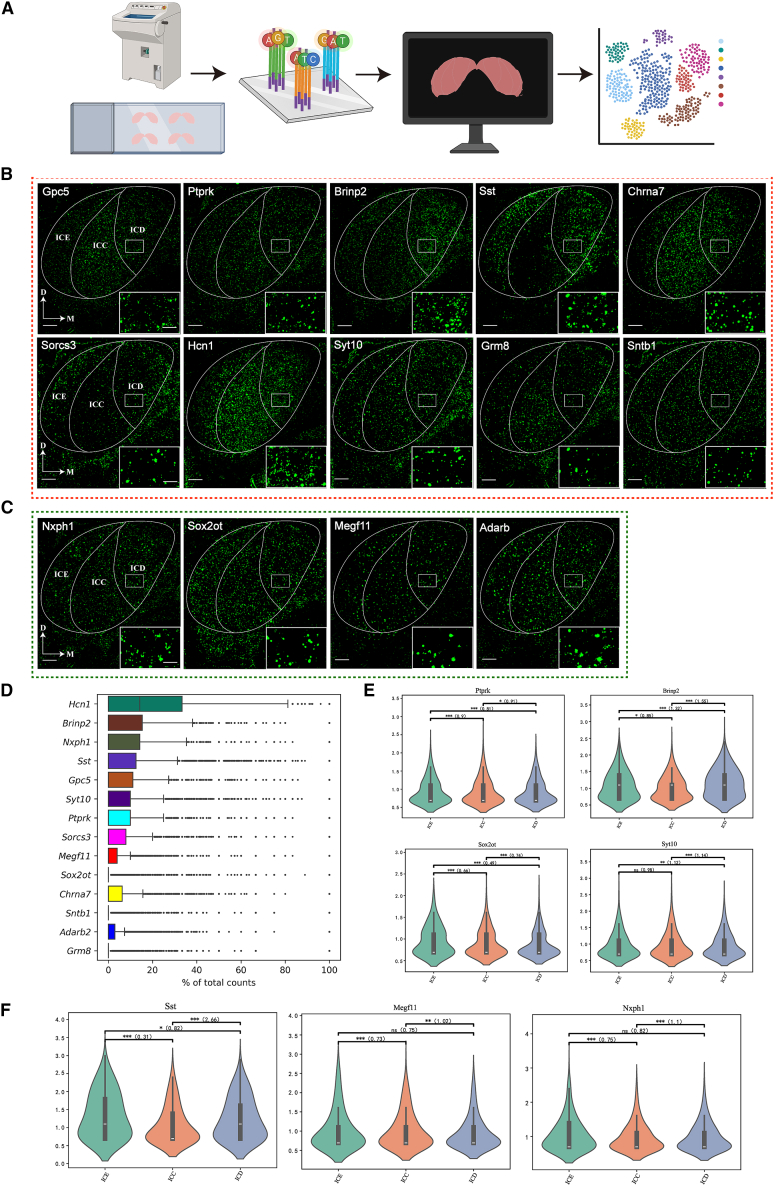
Spatial organization and quantitative analysis of molecularly defined neuronal subtypes in the IC (A) Schematic overview of the ISS workflow, including tissue sectioning, probe hybridization, signal amplification, imaging, and downstream spatial analysis. (B) Spatial localization of excitatory subtype markers (EN1-EN10) across IC subregions (central nucleus, ICC; dorsal cortex, ICD; external cortex, ICE). Insets (lower right) show 2× magnified views of selected regions to highlight transcript localization at higher resolution. Scale bars: 200 μm (main panels); 100 μm (insets). (C) Spatial localization of inhibitory subtype markers (IN1-IN4) across IC subregions. Insets (lower right) show 2× magnified views of selected regions. Anatomical boundaries (ICC, ICD, and ICE), orientation (dorsal, D; medial, M) are indicated in spatial maps. Scale bars: 200 μm (main panels); 100 μm (insets). (D) Quantification of transcript abundance for all subtype-specific markers across IC sections. Expression levels are calculated based on transcript counts and shown as the proportion of total detected transcripts per marker. (E) Violin plots showing markers enriched in the ICD, including *Ptprk*, *Brinp2*, *Sox2ot*, and *Syt10*. (F) Violin plots showing markers enriched in the ICE, including *Sst*, *Megf11*, and *Nxph1*. Statistical comparisons were performed using Mann-Whitney U tests with FDR correction. ns, adjusted *p* ≥ 0.05; ∗ adjusted *p* < 0.05; ∗∗ adjusted *p* < 0.01; ∗∗∗ adjusted *p* < 0.001.

To validate the anatomical organization of transcriptionally defined neuronal subtypes, we performed ISS targeting 16 genes (see Table 1 for probe sequences), including subtype-specific markers for all 14 neuronal clusters (EN1-EN10 and IN1-IN4), together with *Slc17a6* (*Vglut2*) and *Gad2* as pan-excitatory and pan-inhibitory controls (Figure 4A; see STAR Methods for gene selection criteria). Spatial expression data were registered to a reference mouse brain atlas, enabling assignment of signals to IC subregions, including the ICC, ICD, and ICE.

**Table 1:** Probe target sequences used for *in situ* sequencing

Gene	Target sequence (5' → 3′)
Gpc5	CCTACGACGTTGAACACGTGCTCCTGAACTTCCACTTGCT
Gpc5	TTACGGGGGAGATCACCCAAGCCCAACAAGTGGGAGCTCC
Gpc5	TGGCCGCACATTCTTGCAGGCGCTTAATCTGGGCATTGAG
Gpc5	CGCTGTCTCCTTCTCCTGGCTCTCCTTGGATCTACCC
Gpc5	GCTCGGCAAGATTTGCAGCAGGTGCTTCAGACATCC
Gpc5	ACACCTACCGGAACATGGCCTTGGAGGCTGCTGCTT
Ptprk	AGCTACACCCACATCGAACGAAGCGTGAAGCAGGGGCCAT
Ptprk	GTCGTGATTGGCTTCGGGCTGAACTAGCTGTGAGCACCTT
Ptprk	GACAGAGTGGTGAAAATCGCGGGCATCAGTGCTGGCATCC
Ptprk	CGCTGCGTAACTCAGTCAGAACGAGGTTCTGGGGTTTCCA
Ptprk	TTGGCCAGTTCTCAGCAGGTGGCTGTACTTTTGATGATG
Ptprk	TGATACTGCAGGTGGAAAAATGGCAAGAGGAATGTGAAG
Brinp2	AGGCTCCCCAGTGACCGGTTCCTGAACTCCACAGCCATCT
Brinp2	CGCCCCAGAGTTTGTCCGCAATATCCGCCTCCTTGGAAGG
Brinp2	CGGGTGAACCAACTCTCTCCCCCTGGCAAAGTCCGGCTCG
Brinp2	GGAGTATGCTGATTTCATGGAGCGTTACCGCCAGGG
Brinp2	GGTATCCCTGTAGTTGGGGGCACTGGGAACAGCTCCG
Brinp2	GGCTGGTGCATGTGATGTTGGCCTTGTCCTTGCAG
Chrna7	GTACACACCCCTCTGATGGGGACCCCGACCTGGCCAAGAT
Chrna7	GGTTTCTGCGCATGAAGAGGCCGGGAGAGGACAAGGTGCG
Chrna7	GTGTCCCTGCAAGGCGAGTTCCAGAGGAGGCTGT
Chrna7	ACAGATCACTATTTGCAGTGGAACATGTCTGAGTACCC
Chrna7	CACACCAACGTCTTGGTGAATGCATCTGGGCATTGC
Chrna7	GTACTTCGCCAGCACCATGATCATCGTGGGCCTCT
Sorcs3	AGGAAGCCGTCCTCACACCAAGGGTTCGCGAGAGGAGGTG
Sorcs3	CGGGCATTCCATCTCCTAGCCAGGCGGGCAGTGCGAGGAG
Sorcs3	CAATGTCTGGTTCCTAGACTGGGGTGGTGCTCTGGTGG
Sorcs3	AAGCGAGCTCTGATTAAAGTGACCAGTGTCCCAGAAGA
Sorcs3	AATGCGCTTAATCAAAACTTGGTACAGTTTGAGCTGAA
Sorcs3	GTATTCGTCGGCTTGGCTGTGTTTCTGATCTACAAGTT
Hcn1	AACGCCGATCCCAATTTCGTGACGGCCATGCTGAGCAAGC
Hcn1	ATTATCCATCCGTACAGTGACTTCAGGTTTTATTGGGAT
Hcn1	ACGTGGCATCCGATACTGTTTTCCTGTTGGACTTAATCAT
Hcn1	AGAAAGGGATGGACTCAGAAGTTTACAAGACAGCCAGAG
Hcn1	CCTCATTGGCATGATGCTGCTTCTGTGCCACTGGGA
Hcn1	GGACTCTTCAAGGAGGCAGTATCAAGAGAAGTATAAGCAA
Sntb1	AGGGGTGCCACAATTCAGCGGAGCTCACTGCCGAAATCAC
Sntb1	GACCCAAGCCCTGTATGTGGGCGATGCCATCCTGTCGGTG
Sntb1	AAACCAGAAGAGGGGCGTGAAGGTGCTGAAGCAGGAG
Sntb1	CTTCTCCTTCCATAGAGACCGGAAAAGCATCCCCCTC
Sntb1	GACCTTGGCTGACCCTGAAAACAGGCAGCTAGAAATTC
Sntb1	TTCCAACGCTGGTGACTTGCTGACCCGTGTGGTTG
Adarb2	GGGCTGGTTGTCAACGACTGTCATGCTGAGATCGTGGCGA
Adarb2	TCGGCCTGACATCTGTGTATGCCCGCCACAAAACACTGGC
Adarb2	GCATGAGCTGAAGCCTGGTCTGCAGTACCGAATGG
Adarb2	GCAGTGGCTGTAGAAGTGAACGGCCTCACATTTGAAG
Adarb2	GCCTGTGTACCTCCACAGCATCATTGTGGGCAGCC
Adarb2	CATGGTAGGCTAAGCACACGGATCCCCAGCCATG
Gad2	GCCCCAAAGGGGATGTCAACTACGCGTTTCTGCACGCAAC
Gad2	CCTTCGGATCTGAAGATGGCTCTGCGGATCCTGAGAA
Gad2	ACGTGGTGAAAAGTTTCGATAGATCAACTAAAGTGATCG
Gad2	TTGGGAATTGGCAGACCAACCGCAAAATCTGGAGGAAA
Gad2	TGGCGATGGAATCTTTTCTCCTGGTGGCGCCATCTC
Gad2	ATGGGAAGCCTCAACACACAAATGTCTGCTTCTGGTTTG
Grm8	GAAAGCGCTCAACCTCTTGCCCTTGTTTCTTCCTTTTG
Grm8	GAGTATGCGCATTCCATCCGCCTGGATGGGGACATCA
Grm8	GGCATCCACAGACTTGAGGCCATGCTTTATGCAATCGAC
Grm8	CAAGCCCGACAAGATTTCTGGTGTCATAGGTGCTGCAG
Grm8	AGATCTCAAGGGAGATTGGTGGTGTTTGCATTGCTCAA
Grm8	AGAGGGGTTCCAGAAACGTTCAATGAAGCCAAACCTAT
Megf11	GAAGACCCCAATGTGTGTAGCCACTGGGAGAGCTATG
Megf11	CAGCTGGATACATGGGGGACAGGTGTCAAGAAGAATGT
Megf11	AGTGTGAGCCTGGCTACAAGGGCCCTAGCTGCCAG
Megf11	TGCAGGGACCTATGGTCCCAACTGTTCATCTGTATGTA
Megf11	GCAGGACTGTGCCCAGCTCTGTTCCTGTGCCAACA
Megf11	GCGGCAGAAAGAGAAAGGCCGTGACCTGGCTCCCCG
Nxph1	ATGGTGGGAAATCGGAACTACTGAAGTCAGGGAGCAG
Nxph1	CAACATCAAAACAGTGAAGCTAAACCTGTTGATAACTGGGA
Nxph1	TCGTGGAATTCGATTTGGCACAACAAACCGTGATTGATG
Nxph1	GCTCTGCTCTAAGCCCTTCAAGGTGATCTGTATTTACA
Nxph1	ATGCCCTGACTACAACTATCACAGTGACACACCTTACTT
Nxph1	AGTATGAGAAGGTTGACAAGGCCACCAAGAACACACTC
Sst	CTCGGACCCCAGACTCCGTCAGTTTCTGCAGAAGT
Sst	ACCGGGAAACAGGAACTGGCCAAGTACTTCTTGGCA
Sst	CTGCTGTCCGAGCCCAACCAGACAGAGAATGATGCC
Sst	TGGAGCCCGAGGATTTGCCCCAGGCAGCTGAGCAG
Sst	GGAGCTGCAGAGGTCTGCCAACTCGAACCCAGCAAT
Sst	AACGCAAAGCTGGCTGCAAGAACTTCTTCTGGAAGA
Syt10	CGCAAGGAGGACGGTGTGAGCAGCCTGTGCCAGAA
Syt10	TTGCTGGTTGTCTCCCTTTTTGTCTTCTGGAAGTTGTGCT
Syt10	ATTGAGCCTGCAATAAAAATCAGCCACACATCCCCCGAC
Syt10	TGCACCGGAAGACTTTAAACCCTCTGTTTGATGAGCTAT
Syt10	ATCTCTCCAGGGAAGCCACAGTATGGAAAGATATCCACT
Syt10	AGGTCAGCCTGTGCATTGCAGTCATGGATTACGACAGGG
Slc17a6(Vglut2)	CTCGGACAGATCTACAGGGTGCTGGAGAAGAAGCAGG
Slc17a6(Vglut2)	GCTGATCCCATCTGCAGCCAGAGTGCATTATGGATGTG
Slc17a6(Vglut2)	TGCAATGCCCTTAGCTGGTATCCTTGTGCAGTACACTGG
Slc17a6(Vglut2)	GAAGGTGCGCAAGACGCGTACACCTATAAGGACCGA
Slc17a6(Vglut2)	AGCATTGGAGAGAGCGCAAATCTGCTAGGTGCAATGGA
Slc17a6(Vglut2)	GTTTGCCCTATCATTGTTGGTGCAATGACAAAGAATAAGT
Sox2ot	CACTCTCCTGCTGGCTCTCTCTGTCCACACTTAGCTGG
Sox2ot	AGTGGCCATCCATGGGATGAGTGAAAGCCTCTTTCTAT
Sox2ot	CAAACTGCTACAAGACAACACCCTGATCTGGCATGGTCG
Sox2ot	AAATCGGAAAATCTCTGCAGCTGGTACACGCAAAAGAGA
Sox2ot	AATGAGCATTTGAAGGTTTTAAAAGCTTCAGTATTTATTTTG
Sox2ot	CGGAAGGACCATGTGAGCTCATCCCAGATGATGGGTGG

Spatial maps revealed distinct distribution patterns for excitatory and inhibitory subtype markers (Figures 4B and 4C). Quantification of overall transcript abundance across all regions (Figure 4D) showed that markers such as *Hcn1*, *Brinp2*, *Nxph1*, and *Sst* were highly expressed, whereas *Sntb1* (EN10), *Adarb2* (IN4), and *Grm8* (EN9) exhibited lower transcript abundance, consistent with their relative representation in the snRNA-seq dataset.

To quantitatively assess spatial organization, we performed region-wise transcript count analysis by counting detected transcripts for each marker within atlas-defined ICC, ICD, and ICE regions, followed by false discovery rate (FDR) correction for multiple comparisons. Distinct region-specific enrichment patterns were observed. ICC-enriched markers (*Chrna7*, *Gpc5*, *Hcn1*, and *Sorcs3*) showed significantly higher transcript counts in ICC compared to ICD and ICE (*p* < 0.001), with *Chrna7* exhibiting a 2.44-fold increase over ICE and *Hcn1* a 1.68-fold increase (Figure S2A). ICD-enriched markers (*Ptprk*, *Brinp2*, *Sox2ot*, and *Syt10*) were preferentially expressed in ICD, with *Brinp2* showing a 1.55-fold increase over ICC and 1.32-fold over ICE (*p* < 0.001) (Figure 4E). ICE-enriched markers included *Sst*, which displayed the highest transcript levels in ICE (3.23-fold vs. ICC, *p* < 0.001; 1.22-fold vs. ICD, *p* < 0.05), as well as *Megf11* and *Nxph1*, which were elevated in ICE relative to ICC but showed comparable levels between ICE and ICD (Figure 4F). In contrast, broadly distributed markers (*Adarb2*, *Sntb1*, and *Grm8*) exhibited no significant regional differences (*p* > 0.05) (Figure S2B).

Together, these results demonstrate that excitatory neuronal subtypes exhibit pronounced and region-specific spatial enrichment across IC subdivisions, whereas inhibitory subtypes are more broadly distributed with weaker regional specificity.

### *Sst*^+^ neurons form molecularly and spatially distinct subpopulations in the IC

Building on the identification of *Sst*^+^ neurons in the IC, we next investigated their neurotransmitter identity, spatial distribution, and transcriptional heterogeneity.

In contrast to the region-level transcript count analysis used for spatial enrichment, we used segmentation-based single-cell analysis specifically to assess co-localization between *Sst* and the inhibitory marker *Gad2* (Figure S3A). This approach enables single-cell-level quantification of gene co-expression and avoids potential bias from transcript-level overlap.

**Figure 5:**
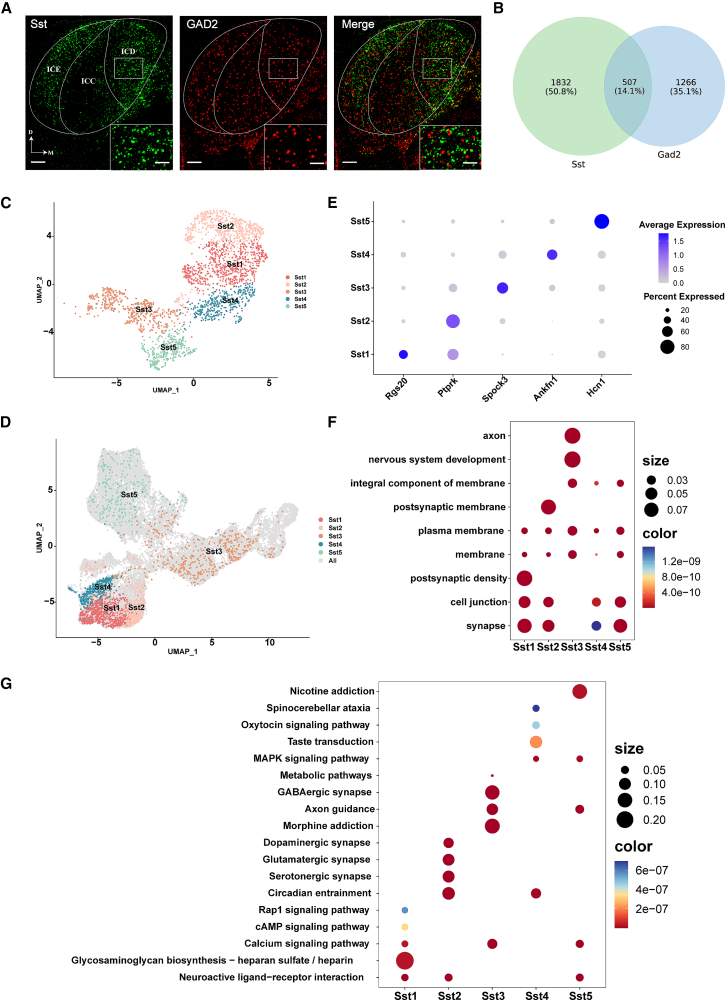
Molecular heterogeneity and neurotransmitter identity of *Sst*^+^ neuronal subtypes in the IC (A) Representative ISS images showing the spatial distribution and co-localization of *Sst* (green) and *Gad2* (red) transcripts in the IC. Merged images reveal that most *Sst*^+^ cells lack *Gad2* signal, while a subset of cells exhibits clear co-expression. Insets (bottom right) show 2× magnified views of the boxed regions. IC subregions (ICC, ICD, and ICE), orientation (D, dorsal; M, medial) are indicated. Scale bars: 200 μm (main images) and 100 μm (insets). (B) Quantitative analysis of *Sst* and *Gad2* co-expression based on cell segmentation and single-cell transcript assignment. Venn diagram showing proportions of *Sst*-only (50.8%), *Gad2*-only (35.1%), and *Sst*^+^/*Gad2*^+^ double-positive cells (14.1%) across the entire IC. (C) UMAP visualization of *Sst*^+^ neurons after sub-clustering, revealing five transcriptionally distinct subtypes (Sst1-Sst5). (D) Projection of *Sst* subtypes onto the global neuronal UMAP, illustrating their distribution across excitatory and inhibitory clusters. (E) Dot plot showing representative subtype-enriched genes for each *Sst* subtype. Dot size represents the percentage of cells expressing the gene, and color indicates average expression level. (F) Gene ontology (GO) enrichment analysis of *Sst* subtypes, highlighting subtype-specific functional categories related to synaptic organization, membrane components, and neuronal development. (G) KEGG pathway enrichment analysis of *Sst* subtypes, revealing distinct pathway associations across subtypes, including neurotransmitter signaling and axon guidance pathways.

ISS imaging revealed that the majority of *Sst*^+^ neurons lacked *Gad2* signal, while a subset of cells showed clear co-expression (Figure 5A). Quantitative analysis at the single-cell level demonstrated that, across the entire IC, *Sst*-only cells accounted for 50.8%, *Gad2*-only cells for 35.1%, and *Sst*^+^/*Gad2*^+^ double-positive cells for 14.1% (Figure 5B). Subregion-specific analysis further revealed pronounced spatial heterogeneity (Figure S3B). After normalization to the total number of *Sst*- and/or *Gad2*-positive cells within each subregion, the proportion of *Sst*-*Gad2* double-positive cells increased progressively from ICC (7.9%) to ICD (14.7%) and was highest in ICE (18.1%), indicating that the inhibitory component of the *Sst*^+^ population is regionally enriched. Consistent with this pattern, *Sst* transcript signals were preferentially enriched in the ICD and ICE subregions.

To resolve transcriptional heterogeneity within the *Sst*^+^ population, we extracted all *Sst*-expressing neurons and performed sub-clustering, identifying five transcriptionally distinct subtypes (Sst1-Sst5) (Figure 5C). Mapping these subtypes back to the global neuronal clusters revealed distinct compositional patterns (Figure 5D). Sst1, Sst2, and Sst4 were almost exclusively derived from excitatory populations, whereas Sst3 exhibited a mixed composition, with 64.8% excitatory and 35.2% inhibitory origin. Sst5 was predominantly excitatory with a minor inhibitory contribution (∼5%) (Figure S3C). These results are consistent with the ISS-based co-localization analysis, supporting the presence of a minor inhibitory *Sst*^+^ subpopulation.

We next characterized subtype-specific molecular signatures. Representative marker genes identified by differential expression analysis (Figure 5E) included *Rgs20* (Sst1), *Ptprk* (Sst2), *Spock3* (Sst3), *Ankfn1* (Sst4), and *Hcn1* (Sst5). To provide a comprehensive view of subtype-specific transcriptional programs, we further generated volcano plots for each Sst subtype (Figure S3D), highlighting a broader set of significantly enriched genes. These results confirm that each subtype is defined by a distinct transcriptional profile rather than a single marker gene.

Functional enrichment analysis revealed both shared and subtype-specific features. Gene ontology (GO) analysis showed that all *Sst* subtypes were associated with synaptic and membrane-related processes, with distinct enrichment patterns across subtypes (Figure 5F). KEGG pathway analysis further demonstrated subtype-specific functional associations (Figure 5G). For example, Sst2 was enriched in dopaminergic, glutamatergic, and serotonergic synapse pathways, whereas Sst3 showed enrichment in GABAergic synapse and axon guidance pathways, consistent with its partial inhibitory composition. Sst5 was associated with nicotine addiction-related pathways, suggesting subtype-associated molecular differences that warrant future functional investigation.

Collectively, these results demonstrate that *Sst*^+^ neurons in the IC are predominantly excitatory but include a regionally enriched inhibitory component, and can be further resolved into five transcriptionally distinct subtypes with subtype-specific molecular features. The integration of segmentation-based co-localization analysis, spatial mapping, and transcriptomic profiling provides a comprehensive framework linking molecular identity, neurotransmitter phenotype, and anatomical organization within this neuronal population.

## Discussion

The IC serves as a critical hub for integrating ascending and descending auditory information to support complex behaviors. Anatomically, the IC can be delineated into three major subregions—ICC, ICD, and ICE, distinguished by histological markers such as cytochrome oxidase^39^ or nitric oxide synthase staining,^40^ each contributing to distinct functional roles. The ICC, a lemniscal region receiving tonotopically organized auditory input, is essential for frequency processing^41^ and sound localization.^42^^,^^43^ In contrast, the non-lemniscal ICD exhibits brain state-dependent neuronal activity characterized by differential modulation of excitatory and inhibitory populations,^44^ while the ICE integrates auditory and vibratory signals^10^ and also contributes to attentional modulation.^45^ It remains unclear whether distinct neuronal subtypes within these subregions underlie their functional specialization. Growing evidence from other auditory nuclei—including the cochlear nucleus,^46^ lateral superior olive,^47^ medial geniculate body,^48^ and auditory cortex^49^—demonstrates that molecularly defined cell types correlate strongly with specific connectivity patterns and functional roles. However, a systematic molecular taxonomy of IC neurons has been lacking, limiting mechanistic understanding of how cellular diversity supports auditory and multisensory processing.

Here, we provide an IC-focused molecular and spatial resource for neuronal diversity by integrating snRNA-seq with ISS. Our analysis identifies 14 transcriptionally distinct neuronal subtypes and reveals that excitatory subtypes show pronounced regional enrichment, whereas inhibitory subtypes are more broadly distributed. In addition, we identify a predominantly excitatory *Sst*^+^ neuronal population enriched in non-lemniscal IC subregions and further resolve this population into five transcriptionally distinct subtypes. Together, these findings link molecular identity with anatomical organization in the auditory midbrain.

### Relationship to the Allen brain cell atlas

An important issue is the relationship between our dataset and the ABC atlas. Our comparison using MapMyCells shows strong agreement with the ABC atlas at the level of broad neuronal classes: Allen glutamatergic IC subclasses mapped predominantly to our excitatory populations, whereas Allen GABAergic subclasses aligned with our inhibitory populations. This concordance supports the robustness of our overall classification.

At the same time, our analysis provides complementary IC-focused information alongside the current public reference. First, because our study focuses specifically on the IC, it provides additional IC-focused subtype information within this structure, yielding 10 excitatory and 4 inhibitory neuronal subtypes. Second, we provide direct spatial validation of these subtypes by ISS. Third, we add region-wise quantitative analyses across ICC, ICD, and ICE, as well as segmentation-based cell-level colocalization analysis for *Sst* and *Gad2*. Thus, rather than duplicating the ABC atlas, our dataset complements it by providing higher IC-specific subtype resolution and direct spatial validation.

Methodological differences may also contribute to differences between the datasets. The ABC atlas is based primarily on single-cell RNA sequencing, whereas our study used snRNA-seq, which is particularly well suited for adult neural tissue and may improve recovery of fragile neuronal populations.^36^^,^^37^ Accordingly, the partial mismatch observed at the subtype level likely reflects both increased biological resolution in our IC-focused dataset and technical differences between the two approaches.

### A molecular taxonomy beyond broad excitation and inhibition

Previous neuroanatomical tracing studies have demonstrated that excitatory and inhibitory neurons in distinct subregions of the IC receive different whole-brain projection patterns,^18^ highlighting the need for refined subclassification within these broad neuronal categories. Our molecular taxonomy addresses this by defining 10 excitatory (EN1-EN10) and 4 inhibitory (IN1-IN4) subtypes, revealing organizational principles that extend beyond conventional neurotransmitter-based classification.

For instance, the expression of *Pv* in both an inhibitory (IN3) and excitatory (EN7, and to a lesser extent EN5) subtypes indicates that this calcium-binding protein marks neurons with convergent physiological properties rather than a single neurotransmitter-defined lineage. This observation is consistent with previous work showing that *Pv* is expressed in both GABAergic and glutamatergic neuron populations in the IC, with dynamic shifts during postnatal development.^29^ It is also compatible with recent developmental evidence from related midbrain structures showing that common progenitor pools can generate both excitatory and inhibitory neuron types.^50^ Together, these findings suggest that transcriptomically defined IC subtypes reflect biologically meaningful divisions that are not fully captured by broad excitatory-inhibitory classification alone.

### Spatially segregated excitatory and diffusely organized inhibitory subtypes in the IC

At the level of broad neurotransmitter classes, excitatory and inhibitory neurons do not show clear spatial segregation across IC subregions, consistent with earlier studies reporting overlapping expression of VGLUT2 and vesicular inhibitory amino acid transporter (VIAAT, functionally comparable to *Gad2*-labeled inhibitory populations) throughout the IC.^51^ Extending this population-level view, our transcriptomically guided spatial analysis demonstrates that molecularly defined excitatory subtypes exhibit distinct topographic distributions (Figures 4B, 4E, 4F, and S2). This finding raises the possibility that transcriptionally discrete subpopulations may be associated with functional differences within the IC. For instance, excitatory subtypes defined by markers such as *Gpc5*, *Chrna7*, and *Hcn1* are spatially restricted to the ICC, a region critical for core auditory processing.^41^ The expression of *Hcn1*, which encodes a hyperpolarization-activated cyclic nucleotide-gated channel, together with *Chrna7*, a nicotinic acetylcholine receptor subunit, suggests that these ICC-localized neurons may represent candidate populations for future studies of temporally precise auditory processing and modulatory control within lemniscal pathways. In contrast, ICD-enriched and ICE-enriched excitatory markers point to additional specialization outside the lemniscal core.

Interestingly, this spatial organization aligns with emerging functional evidence. Previous studies suggest that ICD neurons employ specialized decoding mechanisms for interaural level difference processing with inhibitory neurons showing opponent-channel decoding and excitatory neurons relying more strongly on spatially organized population coding.^52^ The concentration of *Brinp2*^+^ excitatory neurons in the ICD identifies a molecularly defined candidate population for future investigation of spatial coding mechanisms. By contrast, the lack of strong spatial confinement among inhibitory subtypes supports the idea that inhibition in the IC may act as a more diffuse modulatory system spanning multiple subdivisions.

### *Sst*^+^ neurons form a heterogeneous population enriched in non-lemniscal IC

Among the neuronal populations identified here, *Sst*^+^ neurons were of particular interest. In forebrain regions such as cortex^53^ and hippocampus,^54^ somatostatin is classically associated with inhibitory interneurons. In contrast, our data indicates that *Sst*^+^ neurons in the IC are predominantly excitatory, while also containing a minor inhibitory component. This conclusion is supported by two complementary observations. First, in the snRNA-seq dataset, *Sst* expression was enriched mainly in excitatory subtypes EN2 and EN4. Second, segmentation-based single-cell colocalization analysis of ISS data showed that most *Sst*^+^ cells lacked *Gad2*, although a subset of *Sst*^+^/*Gad2*^+^ double-positive cells was consistently detected. Notably, this inhibitory component was not uniformly distributed across the IC. The proportion of *Sst*^+^/*Gad2*^+^ cells increased from ICC to ICD and was highest in ICE, indicating pronounced regional heterogeneity. Thus, the *Sst*^+^ population in the IC should not be viewed as a purely excitatory class, but rather as a predominantly excitatory population with a regionally enriched inhibitory component, especially in higher-order non-lemniscal regions. This pattern is also interesting from a circuit perspective. Our previous tracing study showed that *Sst*^+^ IC neurons selectively innervate the posterior limitans thalamus (POL), and that POL further projects to the tail of the striatum (TS).^30^ The POL is considered a higher-order thalamic region,^55^ and previous studies have implicated the TS in threat-, reward-, and avoidance-related behaviors.^56^^,^^57^^,^^58^ Together, the enrichment of *Sst*^+^ neurons in ICD and ICE and their projection relationship with POL identify this population as a candidate entry point for future studies of higher-order IC circuits and their potential links to auditory-guided behavioral functions.

### Transcriptional heterogeneity within the *Sst*^+^ population

Our sub-clustering analysis further showed that *Sst*^+^ neurons are not homogeneous but instead comprise five transcriptionally distinct subtypes (Sst1-Sst5). Four of these subtypes were almost entirely excitatory in origin, whereas Sst3 contained a substantial inhibitory component and Sst5 included a small inhibitory contribution. These subtype differences provide an important framework for interpreting the mixed neurotransmitter identity of the broader *Sst*^+^ population.

Subtype-enriched marker genes and volcano plots further support the transcriptional distinctness of these populations. Functional enrichment analyses suggest both shared and subtype-specific molecular programs. In particular, Sst2 showed enrichment in multiple neurotransmitter-related synapse pathways, whereas Sst3 was associated with GABAergic synapse and axon guidance terms. Importantly, the GABAergic enrichment observed for Sst3 is most parsimoniously explained by the inhibitory component contained within this subtype, rather than as a unique property of excitatory *Sst* neurons. This interpretation is consistent with both the snRNA-seq mapping results and the ISS-based observation that a minority of *Sst*^+^ cells co-express *Gad2*.

Although these enrichment analyses generate useful hypotheses, they do not directly establish physiological function. The current data therefore supports the existence of transcriptionally distinct *Sst*^+^ subtypes with potentially different circuit roles, but functional interpretation will require direct testing using subtype-specific manipulation, circuit tracing, and electrophysiology.

### Limitations of the study

Our study has several limitations. First, while snRNA-seq and ISS provide an IC-focused molecular and spatial resource for neuronal diversity, these approaches do not directly establish electrophysiological properties, long-range connectivity, circuit function, or behavioral roles. Accordingly, the proposed functional roles of molecularly defined IC subtypes, including those inferred from marker enrichment, spatial localization, and pathway enrichment analyses, should be regarded as hypotheses. Direct circuit tracing, electrophysiological recording, subtype-specific manipulation, calcium imaging, and behavioral assays will be required to determine whether and how these neuronal subtypes contribute to auditory computation, multisensory integration, and auditory-guided behaviors. Second, the spatial resolution of ISS, though informative, may not fully resolve dendritic or subcellular transcript localization. Third, our analysis was performed in adult mice under baseline conditions, developmental dynamics and experience-dependent plasticity remain to be explored. In addition, only male mice were used in this study, so potential sex-dependent differences in IC neuronal composition and spatial organization remain to be investigated. Fourth, our ISS analysis focused on representative mid-level IC sections (bregma −4.79 to −5.19 mm). While these sections contain all three IC subdivisions, we cannot rule out rostral-caudal variations in subtype distribution. However, previous studies have shown that molecularly defined IC neuron populations are largely consistent across the rostral-caudal axis,^30^ suggesting the observed spatial organization is likely generalizable. Fifth, although careful anatomical dissection and contamination control were implemented, low-abundance transcripts potentially arising from ambient RNA or neighboring structures remain a general technical consideration in snRNA-seq experiments, particularly in small midbrain nuclei.

In summary, this work establishes an IC-focused molecular and spatial framework for neuronal diversity in the mouse IC. By integrating snRNA-seq, spatial transcriptomics, region-level quantification, and segmentation-based cell-level colocalization analysis, we show that IC neurons comprise multiple transcriptionally distinct subtypes with non-random spatial organization. In particular, we identify a predominantly excitatory but heterogeneous *Sst*^+^ neuronal population enriched in non-lemniscal IC and resolve it into five transcriptionally distinct subtypes. These findings provide a foundation for future studies of cell-type-specific circuit organization and function in the auditory midbrain.

## Resource availability

### Lead contact

Further information and requests for resources and reagents should be directed to and will be fulfilled by the lead contact, Huaizhang Shi (huaizhangshi@163.com).

### Materials availability

This study did not generate new unique reagents.

### Data and code availability

• Data: The raw sequencing data reported in this paper have been deposited in the NCBI Sequence Read Archive under BioProject accession number PRJNA1395172. The data are publicly accessible at: https://trace.ncbi.nlm.nih.gov/Traces/?view=run_browser&acc=SRR36629270&display=data-access. All other data will be shared by the lead contact upon request.
• Code: This paper does not report original code. All software used is described in the key resources table and STAR Methods.
• Other items: Any additional information required to reanalyze the data reported in this paper is available from the lead contact upon request.

## Acknowledgments

M.L. receives funding from National Natural Science Foundation of China Young Scientists Fund (82401346), open research fund (gect-2025-Q01) of Shanghai Key Laboratory of Gene Editing and Cell Therapy for Rare Diseases, Postdoctoral Program in Heilongjiang Province (LBH-Z23220) and The First Affiliated Hospital of Harbin Medical University Scientific Research Innovation Fund (2024B08). T.Z. receives funding from National Key Research and Development Program of China (2023YFC2412105) and Key Research and Development Program of Heilongjiang Province (2023ZX06C07).

## Author contributions

Investigation, data curation, formal analysis, visualization, writing – original draft, M.L., Q.H., and W.F.; investigation, Q.G., T.M., X.L., Q.M., S.X., and J.L.; conceptualization, supervision, writing – review and editing, H.S. and T.Z. validation, writing – review and editing, all authors.

## Declaration of interests

The authors declare no competing interests.

## STAR★Methods

### Key resources table

**Table:** 

REAGENT or RESOURCE	SOURCE	IDENTIFIER
**Deposited data**		

Raw snRNA-seq data	This paper	NCBI SRA: PRJNA1395172

**Experimental models: organisms/strains**		

Mouse: C57BL/6, male, 2 months old	Shu Daqiang (Chengdu) Biotechnology Co., Ltd.	N/A

**Oligonucleotides**		

ISS padlock probes and primers for 16 genes	This paper	See Table 1

**Software and algorithms**		

Illumina bcl2fastq	Illumina	v5.0.1
ImageFlow	Imazen	v0.1.4
Cell Ranger	10x Genomics	v7.2.0
Seurat	Satija Lab	v4.4.0
DoubletFinder	McGinnis et al.	v2.0.4
clusterProfiler	Bioconductor	v4.12.6
Cellpose	Stringer and Pachitariu	v3.0
MapMyCells	Allen Institute	https://portal.brain-map.org/atlases-and-data/bkp/mapmycells
OmicStudio	LC-Bio Technology	https://www.omicstudio.cn/cell

**Critical commercial assays/kits**		

Chromium Next GEM Single Cell 3′ GEM, Library & Gel Bead Kit v3	10x Genomics	N/A
Chromium Next GEM Chip G Single Cell Kit	10x Genomics	N/A
Single Index Kit T Set A	10x Genomics	N/A
Debris Removal Kit	Miltenyi Biotec	130-109-398

**Chemicals, peptides, and recombinant proteins**		

Phi29 DNA polymerase	Vazyme	N106-01
T4 Polynucleotide Kinase	Vazyme	N102-47201
T4 DNA Ligase	Thermo Fisher Scientific	EL0012
Pepsin	Sigma	P0525000

**Other**		

Chromium Single Cell Controller	10x Genomics	N/A
NovaSeq 6000	Illumina	N/A
Leica THUNDER Imaging System	Leica	N/A

### Experimental model and study participant details

#### Animals

All experiments used male wild-type C57BL/6 mice (17–21 g) aged 2 months, which were purchased from Shu Daqiang (Chengdu) Biotechnology Co., Ltd. Only male mice were used in this study. Therefore, potential sex-dependent differences were not assessed and the findings may not be generalizable to females. Mice were housed under standard conditions, with five animals per cage. They had free access to food and water and were maintained on a 12-h light/dark cycle (lights on from 08:00 to 20:00). For snRNA-seq, mice were deeply anesthetized by intraperitoneal injection of 2,2,2-tribromoethanol (Avertin; 200 mg/kg body weight) and decapitated. For ISS experiments, mice were deeply anesthetized before transcardial perfusion with PBS followed by 4% paraformaldehyde. All tissue samples were surgically collected, immediately snap-frozen in liquid nitrogen, and stored at −80°C until further processing. All animal procedures were approved by the Ethics Committee of the First Affiliated Hospital of Harbin Medical University (approval no. 2023092) and were conducted in accordance with institutional guidelines and relevant ethical regulations.

### Method details

#### IC dissection and tissue collection for snRNA-seq

To ensure precise isolation of the IC without contamination from adjacent structures, dissections were performed according to anatomical landmarks described in *Paxinos and Franklin’s The Mouse Brain in Stereotaxic Coordinates*.^59^ Following decapitation, brains were rapidly removed and placed in an ice-cold Petri dish. The IC, located on the dorsal aspect of the midbrain beneath the superior colliculus, was identified under a dissection microscope and carefully isolated along its anatomical borders after removal of the meninges. Samples showing visible contamination from adjacent structures, including the superior colliculus, cerebellum, or periaqueductal gray, were excluded. To ensure consistency, dissections were performed by three researchers familiar with IC anatomy, and each sample was verified by at least two independent observers.

To obtain sufficient nuclei for library preparation, one unilateral IC from each mouse was used. Unilateral IC tissue from three mice was pooled as one sample. In total, three independent pooled samples were prepared (*n* = 9 mice total). Dissected tissues were snap-frozen in liquid nitrogen and stored at −80°C until nuclei isolation.

#### Nuclei isolation, library preparation, and sequencing

Nuclei were isolated from pooled IC tissue. Briefly, tissue was cut into small pieces (∼0.1 cm) and placed in a mixture of 1 mL lysis buffer and 1 mL phosphate-buffered saline (PBS). After two washes by centrifugation in PBS, 2 mL of pre-chilled lysis buffer was added, and the sample was incubated on ice for 2 min for pre-lysis. The tissue was then homogenized using a Dounce homogenizer (Sigma) with 5–6 gentle strokes. Following incubation on ice for 6 min, 2 mL of pre-chilled 4% bovine Serum Albumin (BSA) was added to dilute the mixture, and the reaction was stopped by pipetting 6–8 times with a Pasteur pipette. The sample was centrifuged at 300 g for 5 min at 4°C, and the supernatant was discarded. The pellet containing nuclei was resuspended in 2 mL of lysis buffer containing 4% BSA and incubated on ice for 3 min. Cell debris was removed using a Miltenyi Biotec kit (Order no. 130-109-398).

After centrifugation (300 g, 5 min, 4°C), nuclei were resuspended in nuclear resuspension buffer (1× PBS, 2% BSA, 0.1% RNase inhibitor, Roche). The suspension was loaded onto a pre-formed sucrose cushion (1.8 M sucrose, 3 mM Mg (OAc)_2_, 1 mM dithiothreitol (DTT), 10 mM Tris (hydroxymethyl) aminomethane HCl (Tris-HCl) in diethyl pyrocarbonate (DEPC)-treated water) in polycarbonate ultracentrifugation tubes and centrifuged at 107,000 g for 2.5 h at 4°C. The supernatant was carefully removed, and the nuclear pellet was resuspended on ice in an RNase-free solution (1× PBS, 0.04% BSA, 0.2 U/μL RNase inhibitor). The nuclear suspension was passed through a 30-μm cell strainer twice to remove aggregates, and nuclei were counted using a Nexcelom Cellometer K2.

A single-nucleus suspension targeting approximately 8,000 nuclei was loaded onto a 10x Genomics Chromium chip. cDNA amplification and library construction were performed according to the manufacturer’s standard protocol. Libraries were sequenced on an Illumina NovaSeq 6000 system (paired-end, 150 bp) by LC-Bio Technology Co., Ltd. (Hangzhou, China).

#### snRNA-seq processing, quality control, clustering, and annotation

Sequencing results were demultiplexed and converted to FASTQ format using Illumina bcl2fastq software (version 5.0.1). Sample demultiplexing, barcode processing, and single-cell 3′ gene counting were performed with Cell Ranger (version 7.2.0), and snRNA-seq data were aligned to the Ensembl genome GRCm39 reference genome. This initial data processing was performed with the assistance of LC-Bio Technology.

Downstream analysis was performed primarily using Seurat (version 4.4.0) in R. The OmicStudio online platform (https://www.omicstudio.cn/cell), which implements Seurat-based workflows, was used for parameter tracking and analysis support. Nuclei with fewer than 500 detected genes or mitochondrial transcript fractions greater than 25% were excluded. The 500-gene threshold was selected based on the empirical distribution of detected genes and was used to remove low-quality nuclei and empty droplets, consistent with widely used standards in the field.^60^ Potential doublets were identified and removed using DoubletFinder (version 2.0.4). Data were normalized using NormalizeData, highly variable genes were identified using FindVariableFeatures, and scaled using ScaleData. Principal component analysis (PCA) was performed on 2,000 highly variable genes. The top 20 principal components (PCs) were used for graph-based clustering (FindNeighbors, FindClusters; resolution = 0.5), and UMAP was used for visualization. Cluster stability was evaluated by varying the number of PCs from 10 to 30; clustering with ≥20 PCs consistently yielded 14 neuronal subtypes. Major cell classes were annotated based on canonical marker genes and further evaluated using SingleR and scCATCH.

Differentially expressed genes for each cluster were identified using FindAllMarkers (Wilcoxon rank-sum test) with the following thresholds: log2 fold change ≥0.26, adjusted *p*-value <0.01, and minimum detection rate (min.pct) ≥ 0.1. Although this permissive log2FC threshold was used for initial screening, the top marker genes for each cluster generally exhibited substantially larger fold changes, supporting their biological relevance as robust subtype markers. Functional enrichment analysis of cluster marker genes was performed using clusterProfiler (version 4.12.6) with mouse annotation from org.Mm.e.g.,.db.

#### Sub-clustering analysis of *sst*-positive neurons

To resolve heterogeneity within the *Sst*-positive population, all neuronal nuclei with detectable *Sst* expression (>0 UMI) were extracted from the snRNA-seq dataset (*n* = 2,337). Highly variable genes were recalculated within this subset, and independent sub-clustering was performed using the same Seurat workflow described above. PCA was performed on 2,000 highly variable genes, and the top 20 PCs were used for clustering and UMAP visualization. The resulting *Sst* subtypes were then projected back onto the global neuronal UMAP to determine their excitatory or inhibitory origins.

#### Tissue preparation for *in situ* sequencing

For ISS experiments, brains from two mice were collected after deep anesthesia and transcardial perfusion with PBS followed by 4% paraformaldehyde (PFA). Brains were post-fixed in 4% PFA for 12 h at 4°C, cryoprotected in 30% sucrose, embedded in OCT, and sectioned coronally at 10 μm using a Leica cryostat.

Eight mid-level IC sections (four per mouse) corresponding to approximately bregma −4.87 to −5.17 mm (based on the Allen Mouse Brain Atlas) were selected. These sections contained all three IC subdivisions: the ICC, ICD and ICE. Sections were stored at −80°C until ISS processing.

For ISS pretreatment, sections were mounted in Secure-Seal hybridization chambers and processed as follows: two rinses in ice-cold DEPC-treated PBS; fixation in 4% PFA for 10 min; three washes in DEPC-treated PBST (PBS supplemented with 0.1% Tween 20 and 0.1 U/mL RNase inhibitor); incubation in pre-chilled methanol at −80°C for 15 min; and digestion with 2 mg/mL pepsin (Sigma, P0525000) in 0.1 M HCl for 90 s at 37°C. Sections were then washed in DEPC-treated PBST and processed for ISS.

#### Gene selection for ISS imaging

The 16 genes selected for ISS analysis were chosen based on the following criteria.

1. Subtype-specific marker genes: for each of the 10 excitatory (EN1–EN10) and 4 inhibitory (IN1–IN4) subtypes identified by snRNA-seq, we selected the most specific marker gene with the highest differential expression (log2FC > 1, adjusted p < 0.01). This yielded 14 subtype-specific markers: *Gpc*5 (EN1), *Ptprk* (EN2), *Brinp2* (EN3), *Sst* (EN4), *Chrna7* (EN5), *Sorcs3* (EN6), *Hcn1* (EN7), *Syt10* (EN8), *Grm8* (EN9), *Sntb1* (EN10), *Nxph1* (IN1), *Sox2ot* (IN2), *Megf11* (IN3), and *Adarb2* (IN4).
2. Pan-neuronal markers: to validate excitatory and inhibitory identity, *Slc17a6* (*Vglut2*) and *Gad2* were included as pan-excitatory and pan-inhibitory markers, respectively.
3. Technical compatibility: all selected genes were verified to be compatible with ISS probe design and to have sufficient expression for reliable detection.

The selected genes and their corresponding subtypes are shown in Figure 2E.

#### Probe design

A set of sixteen genes from the IC was chosen for ISS analysis (see Table 1). For ISS, the probe design was based on padlock probes combined with a rolling circle amplification (RCA) initiator primer to drive target-dependent rolling circle amplification. Furthermore, the probe system comprised detection probes for RCA as well as ISS probes. The methodology for probe design was implemented as described previously.^61^ In brief, the probes were designed based on the following criteria: 1) Two target sites per gene were preferentially located in exons near the 5′ end. 2) The padlock probes were designed with target-complementary ends (12–16 bp) and a middle section containing an anchor primer sequence and a barcode. 3) The RCA initiator primer was designed to have the 5′ end matching the target and the 3′ end complementary to the padlock probe. 4) RCA efficiency was monitored by a Cy3-labeled detection probe (cy3-p4). 5) The ISS probe set consisted of three anchor primers and four query probes, each labeled with a distinct Alexa Fluor dye.

For visualization in Figure 2F, ISS signals from all 10 EN subtype markers (*Gpc5*, *Ptprk*, *Brinp2*, *Sst*, *Chrna7*, *Sorcs3*, *Hcn1*, *Syt10*, *Grm8* and *Sntb1*) were merged into a single green channel, and signals from all 4 IN subtype markers (*Nxph1*, *Sox2ot*, *Megf11*, *Adarb2*) were merged into a separate green channel. These composite images were then overlaid with *Vglut2* (red) or *Gad2* (red) to assess co-localization and validate neurotransmitter identity. Individual marker spatial distributions are shown separately in Figures 4B and 4C. Image processing was performed using ImageJ.^62^

#### *In situ* sequencing

The ISS protocol was adapted from an established method.^61^ Briefly, padlock probes were first phosphorylated at the 5′ end using T4 polynucleotide kinase (Vazyme, N102-472 01). These phosphorylated probes, together with RCA initiator probes—each at a concentration of 30 nM—were then mixed in a hybridization buffer containing 2× Saline Sodium Citrate (SSC; Sangon, B548109), 10% formamide (Sangon, A100606), and 20 mM Reduced Vitamin C (RVC; Beyotime, R0108). The resulting solution was applied onto pretreated brain slices placed in hybridization chambers and incubated overnight at 37°C.Subsequent to hybridization, the samples were subjected to two washes with DEPC-PBSRT and one wash with 4× SSC. A ligase reaction mixture was then introduced into the chambers, followed by a 2-h incubation at 37°C.

After two additional washes with DEPC-PBSTR, RCA reaction was performed for 2 h at 30°C using a solution composed of 1 U/μL Phi29 (Vazyme, N106-01), 1× RCA buffer, 0.25 μM dNTP, 0.2 μg/μL BSA, and 5% glycerol.

Following the RCA step and subsequent washing, the samples were incubated for 2 h at 25°C with a sequencing mixture containing 1× BSA, 0.2 U/μL T4 DNA ligase (ThermoFisher, EL0012), 1× T4 DNA ligase buffer, 1 μM anchor primer, and 1 μM query primer. The samples were then washed thrice with a solution of 10% formamide in 2× SSC, stained with DAPI, and finally imaged using a Leica THUNDER Imaging System with a 20× objective (NA = 0.80).

To enable multiple sequencing rounds, the signal was eradicated after each imaging cycle by two 10-min treatments with a stripping buffer (60% formamide in 2× SSC) at room temperature, prior to the application of the next round’s sequencing mixture.

#### Image registration, candidate spot calling, and decoding

For cross-round image alignment, a standardized procedure was implemented. Initially, all raw images were normalized to a uniform size via the ‘resize’ function in the scikit-image toolkit. Images from the initial sequencing round were assigned as reference templates. Prior to registration, background noise suppression was performed using the white_tophat function, configured with a ball-shaped structural element of size 1. The final co-registration of subsequent rounds to the reference set was executed using the SimpleElastix toolkit, utilizing an “Affine” transformation parameter set.

For candidate spot calling, background noise was first eliminated. Subsequent intensity balancing across channels was achieved by identifying reference points via the “h_maxima” function (scikit-image), where the “h” parameter was calibrated to the 95% quantile of each channel’s intensity distribution. Each channel was normalized according to the mean intensity calculated from its respective reference points. An aggregated mean image was then generated from the vertex of these normalized channels. This composite image served for more complex analyses, and candidate points were designated upon it by re-applying the “h_maxima” function, using the 95% quantile intensity of the aggregated image as the “h” parameter.

For decoding, dual-channel barcode intensities were estimated by combining channel intensities according to the formula: I_ch1, ch2 = √ (I_ch1 × I_ch2). For each spot, a volumetric region of dimensions 5 × 5×3 (x, y, z) was delineated and compressed into a 5 × 5 matrix projection. Within this area, the point of maximum intensity (denoted mmii) served as the reference for calculating p_ij (i ≤ 3, j ≤ 4), representing the average intensity of the surrounding 3 × 3 sub-matrix across all rounds and channels.Candidate spots were eliminated based on two criteria, location at image boundaries or min_i(max_j(p_ij)) ≤ I_threshold. For the remaining spots in each imaging round, the sorted intensity values across channels (VS = {S_1_, S_2_, S_3_, S_4_}) were analyzed. Classification as a single-channel signal required S_1_ > 2 × S_2_; otherwise, the spot was designated as a dual-channel merged signal. The integrated barcodes from all rounds were subsequently matched against a predefined gene codebook to identify genes of interest.

#### Comparison with the Allen Brain Cell Atlas

To compare our IC neuronal taxonomy with a publicly available reference, we performed reference mapping using the ABC Atlas.^35^ Allen IC subclass annotations were projected onto our snRNA-seq dataset using MapMyCells. Cells that could not be confidently assigned to an existing Allen IC subclass were labeled as “NA.” Reference mapping was restricted to IC-related neuronal subclasses in the ABC Atlas and was used for comparative benchmarking of major neuronal classes and subtype resolution, rather than for direct raw-data integration.

### Quantification and statistical analysis

#### Quantitative analysis of regional transcript distribution

IC subregions (ICC, ICD, and ICE) were delineated according to atlas registration and anatomical landmarks. For each marker, detected transcripts were counted within each subregion on each section, and region-wise comparisons were performed using two-tailed Mann–Whitney U tests. Fold change was calculated as the ratio of mean transcript counts between regions. Raw *p*-values were corrected for multiple comparisons using false discovery rate (FDR) correction (α = 0.05). Significance levels were assigned as follows: ns (adjusted *p* ≥ 0.05), ∗ (0.01 ≤ adjusted *p* < 0.05), ∗∗ (0.001 ≤ adjusted *p* < 0.01), and ∗∗∗ (adjusted *p* < 0.001).

Violin plots were generated from log-transformed transcript counts [log (x + 1)] and display median and quartiles. The spatial quantification shown in Figures 4 and S2 was performed at the transcript-count level within atlas-defined anatomical regions and was not based on segmentation-derived single-cell subtype assignment.

#### Cell segmentation and transcript assignment for colocalization analysis

Segmentation-based single-cell analysis was used specifically for *Sst*/*Gad2* colocalization analysis shown in Figures 5 and S3. Cell segmentation was performed using Cellpose v3.0^63^ based on DAPI nuclear staining images with the cyto3 pre-trained model. Segmentation results were manually inspected and corrected using the interactive correction feature to ensure accuracy. Representative segmentation results are shown in Figure S3A.

Following segmentation, transcripts were assigned to individual cells using the KD-tree algorithm implemented in ImageFlow software. This approach converts signal spots and cell boundary coordinates into a spatial index and performs radius-adaptive search for each transcript, generating a single-cell gene expression matrix for downstream co-localization analysis.

#### Colocalization analysis of *Sst* and *Gad2*

For co-localization analysis, cells with more than one assigned transcript for *Sst* or *Gad2* were considered positive for the corresponding gene. Each segmented cell was assigned to ICC, ICD, or ICE according to its centroid position within atlas-registered subregion boundaries. For each subregion and for the entire IC, cells were classified as *Sst*-only, *Gad2*-only, or double-positive, and percentages were calculated relative to the total number of cells positive for either gene. Venn diagrams were generated using matplotlib Venn. Cell counts and percentages were displayed for each category and rounded to one decimal place.

#### Additional resources

This study did not generate additional resources.
