# Supplementary material for: Molecular taxonomy and spatial organization define neuronal subtypes in the mouse inferior colliculus
Source: iScience. 2026 Jun 23;29(7):116484. doi: 10.1016/j.isci.2026.116484 (PMC13320336; doi:10.1016/j.isci.2026.116484)
Supplement: Document S1. Figures S1–S3 [file mmc1.pdf]

iScience, Volume *29*

Supplemental information

Molecular taxonomy and spatial organization define neuronal subtypes in the mouse inferior colliculus

Mengting Liu, Qi Hu, Wenhao Feng, Qi Guo, Tianyu Ma, Xiang Li, Qi Meng, Shijia Xu, Jueqi Li, Tianhong Zhang, and Huaizhang Shi

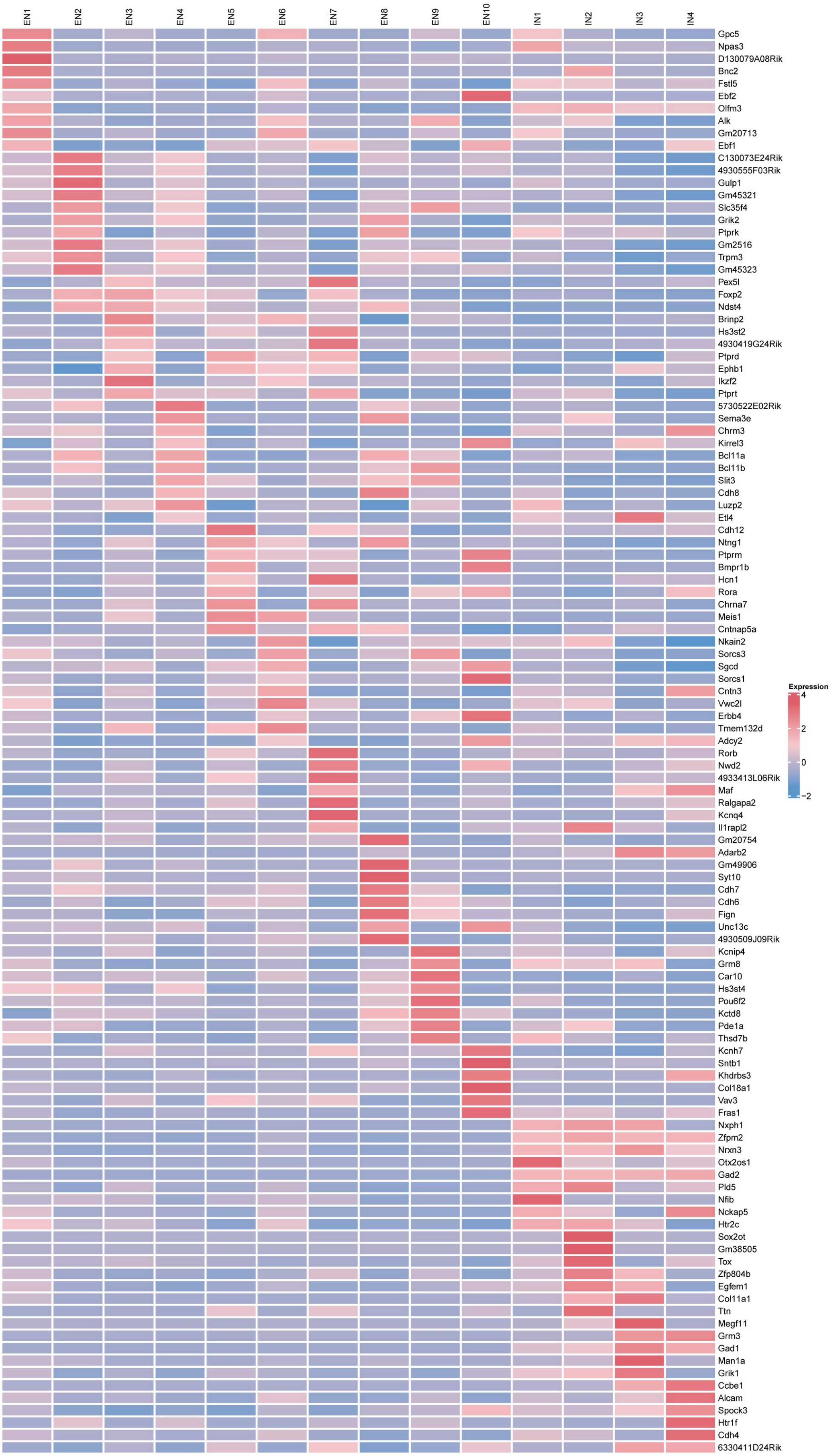
EN1
EN2
EN3
EN4
EN5
EN6
EN7
EN8
EN9
EN10
IN1
IN2
IN3
IN4
Gpc5
Npas3
D130079A08Rik
Bnc2
Fstl5
Ebf2
Olfm3
Alk
Gm20713
Ebf1
C130073E24Rik
4930555F03Rik
Gulp1
Gm45321
Slc35f4
Grik2
Ptprk
Gm2516
Trpm3
Gm45323
Pex5l
Foxp2
Ndst4
Brinp2
Hs3st2
4930419G24Rik
Ptprd
Ephb1
Ikzf2
Ptprt
5730522E02Rik
Sema3e
Chrm3
Kirrel3
Bcl11a
Bcl11b
Slit3
Cdh8
Luzp2
Etl4
Cdh12
Ntng1
Ptprm
Bmpr1b
Hcn1
Rora
Chrna7
Meis1
Cntnap5a
Nkain2
Sorcs3
Sgcd
Sorcs1
Cntn3
Vwc2l
Erbb4
Tmem132d
Adcy2
Rorb
Nwd2
4933413L06Rik
Maf
Ralgapa2
Kcnq4
Il1rapl2
Gm20754
Adarb2
Gm49906
Syt10
Cdh7
Cdh6
Fign
Unc13c
4930509J09Rik
Kcnip4
Grm8
Car10
Hs3st4
Pou6f2
Kctd8
Pde1a
Thsd7b
Kcnh7
Sntb1
Khdrbs3
Col18a1
Vav3
Fras1
Nxph1
Zfpm2
Nrxn3
Otx2os1
Gad2
Pld5
Nfib
Nckap5
Htr2c
Sox2ot
Gm38505
Tox
Zfp804b
Egfem1
Col11a1
Ttn
Megf11
Grm3
Gad1
Man1a
Grik1
Ccbe1
Alcam
Spock3
Htr1f
Cdh4
6330411D24Rik
Expression
4
2
0
−2

Figure S1. Heatmap of subtype-specific gene expression across IC neuronal clusters

Heatmap showing the expression patterns of representative marker genes across the 14 neuronal subtypes (EN1–EN10 and IN1–IN4) identified by snRNA-seq. For each subtype, the top differentially expressed genes were selected based on adjusted p-value and fold change criteria. Rows represent genes and columns represent individual cells (randomly sampled from each cluster). Expression values are scaled (z-score) across cells to highlight relative enrichment patterns. Distinct and subtype-specific gene expression signatures are observed across all clusters, supporting the robustness of the clustering and the transcriptional separation of neuronal subtypes in the IC.

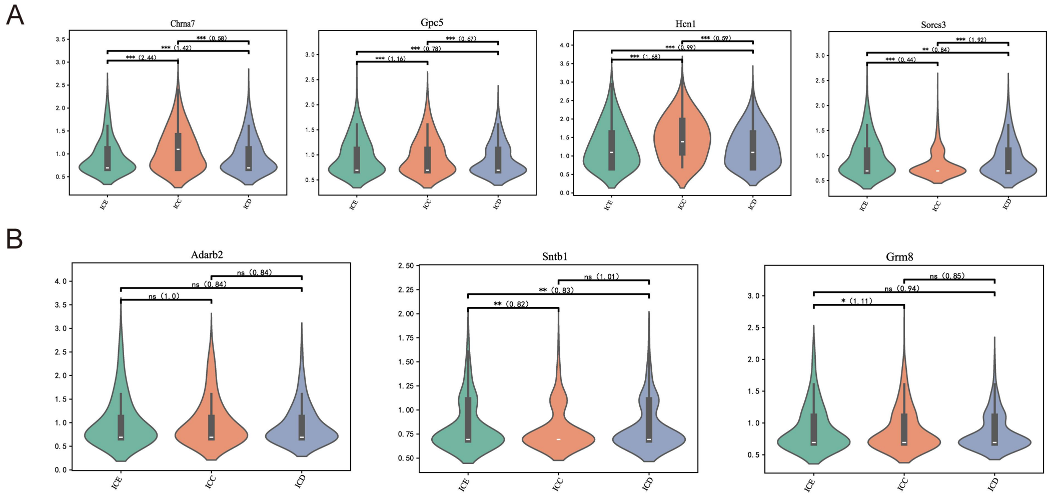

Figure S2. Region-specific and non-specific spatial distribution of subtype markers in the IC.

(A) Violin plots showing markers enriched in the central nucleus of the IC (ICC), including *Chrna7*, *Gpc5*, *Hcn1*, and *Sorcs3*. (B) Violin plots showing markers without significant spatial specificity across IC subregions, including *Adarb2*, *Sntb1*, and *Grm8*. All quantifications are based on region-wise transcript counts. Statistical comparisons were performed using Mann–Whitney U tests with FDR correction. ns, adjusted $p \geqslant 0.05$; * adjusted $p < 0.05$; ** adjusted $p < 0.01$; *** adjusted $p < 0.001$.

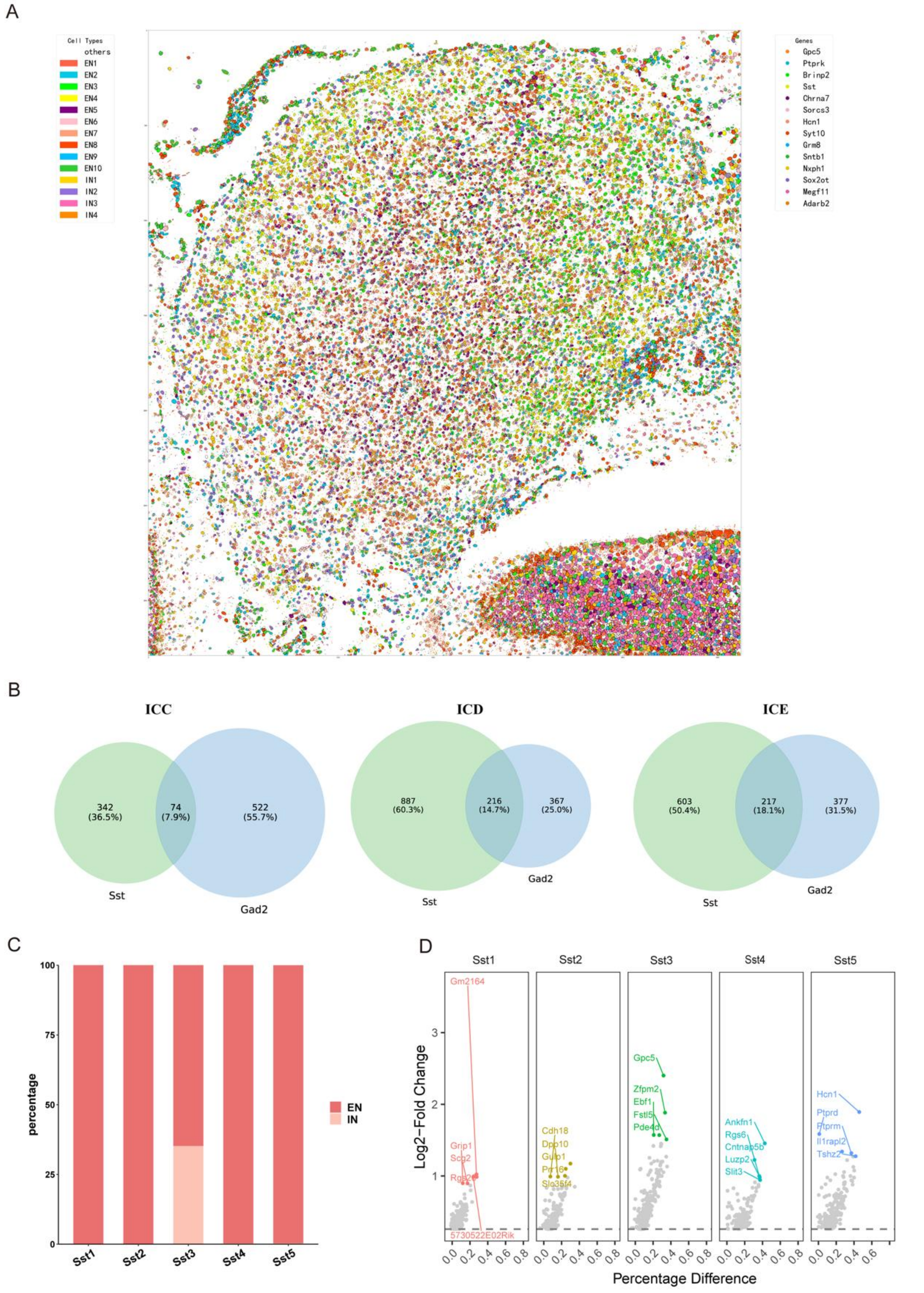

Figure S3. Segmentation-based co-localization analysis and subtype characterization of *Sst*⁺ neurons in the IC.

(A) Representative cell segmentation results based on DAPI staining. Individual nuclei were segmented using Cellpose and manually curated to ensure accurate cell

boundary identification. Segmented cell masks were used for subsequent assignment of transcripts to individual cells and single-cell–level co-localization analysis. (B) Quantitative analysis of *Sst* and *Gad2* co-expression across IC subregions. Venn diagrams show the proportions of *Sst*-only, *Gad2*-only, and *Sst*$^{+}$/*Gad2*$^{+}$ double-positive cells in the central (ICC), dorsal (ICD), and external (ICE) cortices. The proportion of double-positive cells increases from ICC to ICE, indicating region-specific heterogeneity of *Sst*$^{+}$ neuronal populations. (C) Proportion of excitatory (EN) and inhibitory (IN) neuronal origins for each *Sst* subtype. Bar plots show that Sst1, Sst2, Sst4, and Sst5 are predominantly excitatory, whereas Sst3 contains a substantial inhibitory component, consistent with co-localization analysis. (D) Volcano plots showing differentially expressed genes for each *Sst* subtype (Sst1–Sst5). The x-axis represents the difference in expression percentage between the target subtype and other cells, and the y-axis shows log2 fold change. Representative subtype-enriched genes are highlighted, demonstrating distinct transcriptional signatures across *Sst* subtypes.
